# Laboratory or Field Tests for Evaluating Firefighters' Work Capacity?

**DOI:** 10.1371/journal.pone.0091215

**Published:** 2014-03-10

**Authors:** Ann-Sofie Lindberg, Juha Oksa, Christer Malm

**Affiliations:** 1 Sports Medicine Unit, Umeå University, Umeå, Sweden; 2 Winternet, Boden, Sweden; 3 Physical Work Capacity Team, Finnish Institute of Occupational Health, Oulu, Finland; The University of Queensland, Australia

## Abstract

Muscle strength is important for firefighters work capacity. Laboratory tests used for measurements of muscle strength, however, are complicated, expensive and time consuming. The aims of the present study were to investigate correlations between physical capacity within commonly occurring and physically demanding firefighting work tasks and both laboratory and field tests in full time (N = 8) and part-time (N = 10) male firefighters and civilian men (N = 8) and women (N = 12), and also to give recommendations as to which field tests might be useful for evaluating firefighters' physical work capacity. Laboratory tests of isokinetic maximal (IM) and endurance (IE) muscle power and dynamic balance, field tests including maximal and endurance muscle performance, and simulated firefighting work tasks were performed. Correlations with work capacity were analyzed with Spearman's rank correlation coefficient (r_s_). The highest significant (p<0.01) correlations with laboratory and field tests were for *Cutting*: IE trunk extension (r_s_ = 0.72) and maximal hand grip strength (r_s_ = 0.67), for *Stairs*: IE shoulder flexion (r_s_ = −0.81) and barbell shoulder press (r_s_ = −0.77), for *Pulling*: IE shoulder extension (r_s_ = −0.82) and bench press (r_s_ = −0.85), for *Demolition*: IE knee extension (r_s_ = 0.75) and bench press (r_s_ = 0.83), for *Rescue*: IE shoulder flexion (r_s_ = −0.83) and bench press (r_s_ = −0.82), and for the *Terrain* work task: IE trunk flexion (r_s_ = −0.58) and upright barbell row (r_s_ = −0.70). In conclusion, field tests may be used instead of laboratory tests. Maximal hand grip strength, bench press, chin ups, dips, upright barbell row, standing broad jump, and barbell shoulder press were strongly correlated (r_s_≥0.7) with work capacity and are therefore recommended for evaluating firefighters work capacity.

## Introduction

Within several occupations, physical work capacity is important for work performance [1]–[3]. Work capacity has previously been defined as “*physiological capacities in relation to job requirements”* [4] and includes such measures as muscle strength and aerobic power. Work performance is a multi-dimensional and dynamic concept [5] and includes other capacities as well, such as competence, experience and tactic. Firefighting operations include a wide range of work-related activities and movements that occur with varying frequency and intensity [6]–[9]. Firefighters are assumed to be well prepared to perform all associated work tasks, and several work tasks, including carrying equipment up stairs, smoke diving with a breathing apparatus (BA firefighting), and victim rescue are by firefighters rated as physically demanding [7]–[10].

The physical strain imposed on firefighters is multifactorial. Heavy equipment [11]–[13], increased body temperature [14], [15], the use of protective gear [16]–[21] (such as BA), the time passing between turn-outs [22], emotional stress [23], and ergonomics all combine to make many work tasks extremely physically demanding. High levels of aerobic fitness [3], [8] and muscular strength [3], [24] are required to firefighting work tasks. Aerobic work capacity is the most frequently studied and deemed important for firefighters' work performance. Both the absolute (mL·min^−1^) [18], [25]–[27] and the relative (mL·kg^−1^·min^−1^) [8], [27]–[29] maximal aerobic power are of fundamental importance for performance.

Firefighters' muscular strength and endurance are well documented and significant univariate correlations or predictions with work capacity has previously been found [6], [26], [30]–[35]. Maximal grip strength [26], [30], [34], [35], standing broad jump [32], [35], push up endurance [31], [34], [35], 1 repetition maximum (RM) bench press [26], [31], [34], 1 RM squat [31], [34], and shoulder press endurance [30] are some of the simple field tests that have been correlated with firefighters' work capacity. Although a significant correlation between isokinetic knee flexion performance and carrying a stretcher over terrain has been documented among ambulance personnel [1], no study has investigated correlations between advanced laboratory tests and firefighters' work capacity as requested by Barr et al. [3].

There is a lack of research investigating the importance of balance and dynamic stability among firefighters. Punakallio [36] concluded that poor balance reduces the perceived work ability, and that poor dynamic stability increases the risk of slipping and falling [37].

In 2011, the Swedish Fire and Rescue Service were made up of 31% full-time firefighters and 69% part-time firefighters. In accordance with government regulation [38], both groups are required to pass the same tests of physical capacity to be authorized to perform smoke diving. When recruiting firefighters, individual municipalities frequently perform additional physical tests that are not governed by regulations and are not based on scientific studies. These tests might not serve the purpose of selecting the most suitable personnel but instead might discriminate against individuals based on incorrectly designed testing procedures. Specific fitness tests that are accurately correlated with firefighters' work capacity must be developed by using a wide range of fitness tests and work-related outcome variables. Using simple physical tests has been advocated in order for common usage to apply [6], [33] whereas advanced laboratory testing is complicated, expensive and difficult to access.

Therefore, the aims of the present study were a) to investigate correlations between laboratory tests of absolute and relative (scaled to body weight) power, dynamic balance, and commonly occurring and physically demanding firefighting tasks b) to investigate correlations between field tests and commonly occurring and physically demanding firefighting tasks, and c) to give recommendations as to which field tests might be useful for evaluating firefighters' physical work capacity.

## Methods

### Subjects

Forty-two subjects volunteered to participate in this study after receiving written and verbal explanation of the procedure. The 38 subjects completing the study included male full-time firefighters (MFF, N = 8) and male part-time firefighters (MPF, N = 10). In addition, the study included civilian men (CM, N = 8) and civilian women (CW, N = 12) with no previous experience of working as a firefighter. No female firefighters were available to participate in this study. Four subjects decided not to complete the study due to lack of time.

Subjects were recruited from the Fire and Rescue Services in northern Sweden and by notices at Luleå University of Technology and local gyms. All participants signed an informed consent stating their ability to execute all parts of the study and absence of any known diseases affecting physical performance. The same subjects were also included in another study that evaluated aerobic work capacity in firefighters [27].

### Ethics statement

The Research Ethics Committee for Northern Sweden at Umeå University approved the study on September 22, 2009 (Dnr 09-046M), and the study was conducted in accordance with the WMA Declaration of Helsinki – Ethical Principles for Medical Research Involving Human Subjects 2008.

### Study design

Previous studies [39], [40] identified the most physically demanding work tasks that occur the most frequently among Swedish firefighters. The work tasks that qualified for further investigation were based on these studies results [39], [40] and discussions with an expert group from the Swedish Civil Contingencies Agency (SCCA).

Eight laboratory tests and ten field tests investigating muscle performance and dynamic balance, and seven simulated firefighting work tasks were performed in order to select and standardize physical work capacity tests for Swedish firefighters. The tests were executed over seven non-consecutive randomized days with each testing day separated by at least one non-testing day (Table 1).

**Table 1:** Test order.

Test-day	Physical test
**2**	*Field tests*: Maximal hand grip strength, Sit ups, Endurance hand grip, Squat, Bench press
**3**	*Laboratory tests*: Endurance shoulder press, Endurance deadlift. *Field tests*: Chin-ups, Dips
**4**	*Laboratory tests*: Maximal and endurance shoulder flexion and extension, Maximal and endurance knee extension and flexion. *Field test*: Upright barbell row
**5**	*Field test*: Standing broad jump
**6**	*Laboratory tests*: Endurance trunk extension and flexion, Dynamic balance. *Field test*: Barbell shoulder press
**9**	*Work tasks*: Cutting holes in the roof for fire gas ventilation, Carrying hose baskets up stairs, Hose pulling, Demolition at or after a fire, Victim rescue
**10**	*Work tasks*: Vehicle extrication, Carrying hose baskets over terrain

Laboratory tests (isokinetic tests of absolute and relative muscle power, and dynamic balance), field tests, and simulated firefighting work tasks were performed on seven non-consecutive days. Tests of aerobic capacity were also included for the same subject [27].

### Physical tests

Subjects were always dressed in shorts/pants, T-shirt, and training shoes. Additional clothing and equipment used are described below. Every test-day started with an appropriate and standardized 10 minute warm up procedure using an arm ergometer (Monark Exercise AB, Vansbro, Sweden), a rowing machine (Concept II: Concept 2, Inc. Morrisville, VT, USA), and/or a cycle ergometer (Viasprint 150P: Care Fusion, San Diego, CA, USA). Unless otherwise stated, all tests were separated by a five to ten minute rest period, with all subjects having equal resting period.

On the first test day, subjects filled in a health questionnaire to make sure they did not suffer from any known diseases. The mean blood hemoglobin (B-Hb: g·L^−1^) concentration was recorded from duplicate fingertip blood samples (Hemocue AB, Ängelholm, Sweden). Body weight (kg) and standing height (m) were measured with a calibrated scale (SECA 770) and a stadiometer (SECA GmbH & Co. KG, Hamburg, Germany) wearing only shorts and a T-shirt. Body Mass Index (BMI: kg·m^-2^) was calculated.

#### Laboratory tests

Isokinetic concentric tests of maximal muscle force and muscle endurance were performed on a Biodex Multi-Joint System 3 dynamometer™ (Biodex Medical Systems, New York, USA), the protocols used were a combination of the manufacturer's instructions (Biodex medical system: Operation manual) and protocols developed by the authors. The choice of speed and number of repetitions used aimed to mimic the angle velocity achieved during firefighters work performance, thus angular speed and number of repetitions differs for different tests. Data from a filtered output protocol (windowing) was used in which rapid changes in curve data are suppressed but slow changes (attributed to the subject's strength) are not affected, the acceleration and deceleration phase of the repetition are eliminated (Biodex medical system: Operation manual) [41]. Isokinetic testing of muscle performance offers a wide range of measurements, all giving a measure of strength in different ways. Work measures an individual's ability to produce torque within the test's range of motion, and average power is a measure of total work divided by time [41].

Every isokinetic test was preceded by 3 to 6 sub-maximal trial repetitions, and data is presented as the average absolute (W) and relative (scaled to body weight: W·kg^−1^) power of all repetitions (5, 15, or 30 repetitions).

Each of the tests endurance shoulder press, endurance deadlift and maximal and endurance shoulder flexion and extension was preceded by a 10 min (5 min arm cycling at 25 W and 5 min rowing at resistance 7 and the speed 25 pulls·min^−1^) warm up period.

*Endurance shoulder press (overhead)* was performed according to the authors test protocol. In a standing upright position, subjects grasped the horizontal chin bar attachment with a pronated grip, vertically pushed the attachment up to straight arms overhead, and then pulled back to the starting position. One set of 15 RM at an angular speed of 240°·sec^−1^ was performed.

*Endurance deadlift (floor to knee)* was performed according to the authors test protocol. From a standing position, the horizontal bar attachment was grasped with a pronated grip while keeping the back as straight as possible, elbows extended, knees bent to approximately 90° flexion, and the hip joint in a flexed position. The attachment was lifted vertically by extending the knee and hip joint until the torso was in an upright position, then pressing the attachment back to the starting position. One set of 15 RM at an angular speed of 240°·sec^−1^ was performed, and data for the upward movement is presented.

*Maximal and endurance shoulder flexion and extension* were performed according to the manufacturer's instructions (Biodex medical system: Operation manual). The subjects were seated and stabilized with shoulder and waist straps, the seatback angle setting (seatback tilt) was 70°. With the performing arm straight alongside the body (shoulder joint slightly extended), the subjects grasped the bar attachment and lifted their hand upwards (shoulder flexion). The subjects stopped slightly below full shoulder flexion and then pushed their hand back to the start position (shoulder extension). Two bilateral tests were performed including one set of 5 RM at an angular speed of 60°·sec^−1^ followed by one set of 15 RM at an angular speed of 180°·sec^−1^.

*Maximal and endurance knee extension and flexion* was performed according to the manufacturer's instructions (Biodex medical system: Operation manual) after a 10 min (cycling) warm up at 50 W. The subjects were seated (seatback tilt 85°) and stabilized with shoulder, waist and thigh straps. Knee extension was performed from a starting position in which the knee was fully flexed (just before the lower leg touched the chair) to an almost straight knee. Knee flexion was performed back to start position. Two bilateral tests were performed including one set of 5 RM at an angular speed of 60°·sec^−1^ followed by one set of 30 RM at an angular speed of 180°·sec^−1^.

*Endurance trunk extension and flexion* was performed according to the manufacturer's instructions manual (Biodex medical system: Operation manual) after a 10 min (5 min rowing at resistance 7 and the speed 25 pulls·min^−1^, and 5 min of 75 W cycling) warm up period. Subjects were in a semi-standing position, the front of the seat tilted 15°, and the footrest was positioned so that femur was parallel to the seat (knees slightly flexed). With pelvic, femur, and shoulders stabilized with straps, subject performed 15 RM trunk extension and flexion's at 60°·sec^−1^. Extensions were performed from the starting position to an almost supine, horizontal position.

##### Dynamic balance:

The Biodex Balance System SD (Biodex Medical Systems) was used to measure the overall stability index (SI) according to previous standards (Biodex Balance system SD: Operation manual). The tilt of the balance platform is always 0° to 20° and eight possible stability levels (based on platform inertia) can be used from the easiest (Level 8) to the most difficult (Level 1). Without using the supporting rails, the subjects were asked to stand as still as possible on the platform (diameter 0.55 m) while focusing on the target screen placed at eye height. The test was performed for one minute on Level 4. The SI represents the degree of variance of the foot platform displacement, and a lower value indicates better dynamic balance.

#### Field tests

All tests were performed until voluntary exhaustion. Benches, barbells, a Smith machine, dips and chin up equipment (Precor, CL Fitness, Sweden), dumbbells, and free weights (Casall Sport AB, Sweden) were used. The weights of the barbells, dumbbells, and free weights were verified with the SECA scale. Tests performed at a pre-defined speed using a metronome (Korg MA-30 metronome, Korg and Moore, Marburg, Germany), were stopped if the required pace or range of motion could not be maintained despite three verbal encouragements for correction. Only correctly performed exercises were counted.

The maximal hand grip strength, sit-ups, endurance hand grip, squat, and bench press test was preceded by other physical tests [27], and no additional warm up was necessary.

##### Maximal hand grip strength [42]:

The grip size of the force dynamometer (Grip-D: Eleiko sport AB, Halmstad) was individually adjusted to fit the proximal interphalangeal joint of the third finger. In a standing position, with the straight arm alongside the body, and without pressing the hand against the leg, the subjects squeezed the dynamometer as hard as they could. The best of three trials on each hand was registered as the maximal pressure in kg.

##### Sit-ups [43]:

Subjects were lying on their back with their lower legs placed on a box (height 0.40 m) and their hips placed close to the box. With arms crossed over the chest, the upper body was elevated upward at a rate of 50 full lifts per minute (metronome set at 100) until the angulus inferior scapula was in line with a pre-defined marking on the floor then lowered back to start position. The mark was placed 0.6 m from the box for subjects 1.90 m or taller, 0.55 m from the box for those from 1.76 m to1.89 m tall, 0.50 m from the box for those from 1.61 m to 1.75 m tall, and 0.45 m from the box for those 1.60 m or shorter. The number of completed sit-ups was registered.

##### Endurance hand grip:

In a standing position, with an upright posture and straight arms along the body, subjects held a 27.0 kg dumbbell in each hand until exhaustion (dumbbell dropped) and holding time in seconds was registered on each hand.

##### Squat:

Feet were placed slightly wider than shoulder width, and both hands grasped the 22.0 kg barbell that was placed on the shoulders. From a standing, upright posture, 20 full squats per min (metronome set at 40) were performed on a Smith machine to an approximately 90° knee angle, verified by a goniometer prior the test. The number of completed squats was registered.

##### Bench press:

Lying supine on a non-tilted bench, subjects grasped the 30 kg barbell with hands slightly wider than shoulder width. At a rate of 25 full bench presses per min (metronome set at 50), the weight was lowered to the chest and pressed up to straight arms. The number of completed presses was registered.

The chin up and dips test were preceded by physical tests with other warm up.

##### Chin-ups:

Both hands grasped the chin up bar at shoulder width using a pronated grip. Subjects pulled the body from the position of extended elbows until the chin was at the level of the chin up bar. The subjects used a self-selected pace, and the number of completed chin-ups was registered.

##### Dips:

Hands were placed on two supports 0.56 m apart. From the position of straight arms (extended elbow) and feet hanging freely in the air, subjects lowered their body by flexing their elbows until the upper arm was in a horizontal line and then pushed themselves back up. The test was performed at a self-selected pace, and the number of completed dips was registered.

##### Upright barbell row:

In a standing position, the subjects grasped a 7.5 kg EZ-barbell with a pronated grip. The weight was lifted between the spina iliaca anterior superior and the chin at a rate of 30 full lifts per minute (metronome set at 60). The number of completed lifts was registered.

##### Barbell shoulder press:

The test was preceded by physical tests with other warm up [27], and no additional warm up was necessary. In a standing position, the subjects grasped a 7.5 kg EZ-barbell with a pronated grip. The weight was pushed vertically from chin level to straight arms overhead then returned to the starting position at a rate of 25 presses per minute (metronome set at 50). The number of completed presses was registered.

##### Standing broad jump:

The test was performed after a self-selected warm up. Subjects positioned their toes behind the takeoff board and were instructed to jump as far as possible into a sandpit. Arm swing was allowed and the best jump out of three was registered. One subject did not do the test and was excluded from the statistical analysis including standing broad jump.

#### Simulated work tasks

All work tasks below were performed at maximal speed and/or force, without warming up. An extensive description of the method of each work task performed has previously been presented [27] and below is an abbreviated version. Time to complete each work task was recorded.

##### Cutting holes in the roof for fire gas ventilation (Cutting):

A modified 11.0 kg concrete saw (Husqvarna 371 k, St. Olathe, USA) was moved backwards along a 2 m by 2 m square drawn on the floor at a rate of 40 moves per minute. In order to simulate cutting work, the saw was modified with a 5.0 kg weight taped to the front blade; a 0.1 kg weight was attached to a 0.2 m string on the rear in order to move the saw in a constant height from the ground. The total weight of the modified concrete saw was 16.1 kg. The subjects were instructed to keep the 0.1 kg weight in contact with the floor during the whole test. The test was performed until voluntary exhaustion, but with a maximum time of 15 min (not known to the subject prior the test). One CW subject did not perform the work task and was excluded from the statistical analysis including *Cutting*.

A work task course, including *Carrying hose baskets up stairs* (*Stairs*), *Hose pulling (Pulling*), *Demolition at or after a fire* (*Demolition*), and *Victim rescue* (*Rescue*) tasks were performed in sequence with two minutes of active rest (aimed for moving between the stations) between each work task. The subjects were dressed in a fire emergency jacket, gloves, and breathing apparatus (BA, 19.0 kg±0.5 kg). One CW subject did not perform the work task course and was excluded from the statistical analysis including these work tasks.

##### Stairs:

Two hose baskets (each basket adjusted to 16.0 kg) were carried up four floors (a total vertical lift of 13 m) twice with a 60 sec active rest period while walking down. The subjects were instructed to complete the test as fast as possible. The total time of the two laps, excluding the active rest period, was registered. One CW subject was not able to complete the work task and was excluded from the statistical analysis of this test.

##### Pulling:

A rope (length 25 m, diameter 70 mm, and pull resistance at full length of approximately 220 N) was pulled 20 m as fast as possible, using the arms only and without moving the feet.

##### Demolition:

An 8.5 kg EZ-bar was loaded with 3×2.5 kg weights on one end and a 0.25 kg lock placed at both ends of the bar. The end of the bar not loaded with weights was attached to the ceiling, and the attachment point at the EZ-bar was 1.90 m above the floor. A string was attached between the floor and the ceiling, and the loaded end of the EZ-bar was lifted between the 1.40 m and 1.90 m marks at a frequency of 25 lifts·min^-1^ until voluntary exhaustion.

##### Rescue:

A 75 kg rescue doll was pulled backwards over a concrete floor as fast as possible for 30 m using a chest harness to reduce any effect of different grasping or dragging techniques.

##### Vehicle extrication (Vehicle):

An 18.5 kg spreader (Holmatro SP 3240 t; Wennergren Maskin AB, Grimslöv, Sweden) was held with both hands. Five points at three different heights (0.9 m, 1.2 m, and 1.5 m) from the floor were marked on a wall. The front part of the spreader was pressed against each point for 15 s and then moved to the next point in the following pattern: 0.9–0.9–1.2–1.2–1.5–1.2–1.2–0.9–0.9. The test was performed to voluntary exhaustion but with a maximum time of 10 min (not known to the subjects before the test).

##### Carrying hose baskets over terrain (Terrain):

Following a predefined course, the subjects carried two hose baskets (each basket adjusted to 18.7 kg) for a total of 600 m, one basket for 300 m, and walked or ran 700 m without hose baskets. The subjects wore gloves and were instructed to complete the course as fast as possible.

The mean, and the highest HR achieved during the work task course and during the *Terrain* work task was recorded, and the percentage of known maximal heart rate (% HR_max_) was calculated.

### Statistics

Statistical calculations were carried out with SPSS version 20.0 (IBM Corporation, USA). Parametric variables are presented as means ± SD (min–max) and non-parametric variables are presented as median ± Interquartile range (IQR) (min–max) [44]. Data were assumed to be normally distributed if two out of the following three parameters were achieved: skewness and kurtosis ranged within ±2.58 of the standard error, the Shapiro-Wilk's test was >0.05, or the Q-Q Plot was normally distributed upon visual inspection [45]. For two-sided physical tests, the side having the highest individual performance is included in the analysis. Comparisons between subject groups were assessed using either one-way ANOVA with post hoc Bonferroni (when appropriate) correction (for parametric, non-skewed variables) or Kruskal-Wallis and Mann-Whitney U-tests for non-parametric and skewed variables. When significant differences were found with the Kruskal-Wallis test, the Mann-Whitney U-test was carried out using post hoc Bonferroni correction to avoid Type 1 errors and the p-value was divided by the number of paired comparisons. A p-value<0.01 was considered statistically significant for all tests.

All groups were merged in the correlation analyses. Spearman's rank correlation coefficient (r_s_) was used to analyze correlations between dependent and independent variables and data was excluded pairwise. Reference values according to Dusic [46] were used to interpret the Spearman r_s,_ for both positive and negative correlations. Values from 0.9 to 1.0 were considered very strong, from 0.70 to 0.89 were considered strong, from 0.50 to 0.69 were considered moderate, from 0.30 to 0.49 were considered moderate to low, from 0.16 to 0.29 were considered weak to low, and values from 0.00 to 0.15 were considered [46].

## Results

### Subject characteristics

The average body height was significantly different (p<0.0001) between subject groups: the CW group was significantly shorter than the MPF and CM groups. No significant differences were found between subject groups for age (p = 0.156), BMI (p = 0.64) or B-Hb (p = 0.033). The one-way ANOVA test indicated significant differences (p = 0.008) between subject groups for body weight, but no differences were found in the post hoc analyses (Table 2).

**Table 2:** Anthropometric data.

	Firefighters		Civilians	
	Full-time	Part-time	Men	Women
	N = 10	N = 8	N = 8	N = 12
**Age (Year)**	39±9.1	28±4.7	32±11.4	34±10.7
	(28–57)	(24–36)	(22–53)	(20–53)
**Body height (m)**	1.78±0.04 *†	1.81±0.07 †	1.82±0.05 †	1.70±0.07 *
	(171–184)	(173–193)	(173–189)	(159–187)
**Body weight (kg)**	79±4.5	82±14.3	83±9.0	69±10.0
	(70–86)	(70–107)	(67–99)	(53–88)
**BMI (kg·m^−2^)**	25±1.3	25±4.0	25±2.4	24±2.7
	(23–28)	(20–32)	(21–29)	(21–30)
**B-Hemoglobin (g·L^−1^)**	150±13.6	158±11.2	153±13.2	141±8.1
	(130–168)	(142–174)	(128–170)	(125–153)

When significant differences between subjects groups were found with one-way ANOVA, post hoc Bonferroni analysis was carried out. The mean ± standard deviation (min–max) is presented. Subject groups marked with different symbols in rows (*, †) are significantly different (* significantly different from †) (p<0.01). Total subjects N = 38.

### Physical test performance

#### Laboratory tests

All isokinetic tests of absolute and relative maximal and endurance power in the upper body were significantly different between the subject groups (p = 001 for the relative endurance shoulder press test, and p<0.0001 for the other tests). The CW group had lower absolute and relative power compared to firefighters in all these tests, and was equal to the CM group in the relative maximal and endurance shoulder extension test, and in the absolute and relative endurance shoulder press test (Table 3). Groups of men had equal absolute and relative power in all isokinetic tests of the upper body (Table 3).

**Table 3:** Subject group (N = 38) performances in isokinetic concentric tests of maximal and endurance upper body muscle power.

		Firefighters		Civilians	
Test	SM	Full time	Part time	Men	Women
		N = 10	N = 8	N = 8	N = 12
**MAXIMAL MUSCLE POWER**					
**Shoulder flexion**	P	56±7,2 †	57±6.0 †	55±9.0 †	26±8.8 *
**(W)**		(46–67)	(49–67)	(44–68)	(11–42)
**Shoulder flexion**	P	0.71±0.10 †	0.70±0.08 †	0.65±0.08 †	0.37±0.12 *
**(W·kg^−1^)**		(0.59–0.85)	(0.62–0.81)	(0.54–0.79)	(0.14–0.57)
**Shoulder extension**	P	69±11.4 †	70±9.5 †	66±11.6 †	37±10.0 *
**(W)**		(46–89)	(58–88)	(52–84)	(27–57)
**Shoulder extension**	NP	0.88±0.18 †	0.84±0.13 †	0.76±0.10 *†	0.51±0.17 *
**(W·kg^−1^)**		(0.66–1.13)	(0.75–0.99)	(0.67–0.97)	(0.38–0.98)
**ENDURANCE MUSCLE POWER**					
**Shoulder press**	P	1517±149.9 †	1486±376.5 †	1409±469.1 *†	878±227.6 *
**(W)**		(1313–1716)	(813–1806)	(830–2251)	(586–1337)
**Shoulder press**	P	19±1.48 †	18±4.15 †	17±4.96 *†	13±2.82 *
**(W·kg^−1^)**		(16.0–21.0)	(11.6–23.5)	(10.2–26.1)	(8.5–17.7)
**Shoulder flexion**	P	107±11.1 †	101±12.9 3.7†	98±16.6 †	41±17.5 *
**(W)**		(86–126)	(74–115)	(72–118)	(10–71)
**Shoulder flexion**	P	1.4±0.15 †	1.3±0.15 †	1.2±0.13 †	0.6±0.24 *
**(W·kg^−1^)**		(1.1–1.6)	(1.1–1.5)	(1.0–1.4)	(0.1–1.0)
**Shoulder extension**	NP	144±32.9 †	135±31.8 †	124±37.0 †	54±37.0 *
**(W)**		(85–188)	(111–171)	(99–172)	(43–115)
**Shoulder extension**	NP	1.8±0.49 †	1.7±0.30 †	1.5±0.31 *†	0.9±0.44 *
**(W·kg^−1^)**		(1.2–2.4)	(1.6–1.9)	(1.3–1.7)	(0.6–2.1)

Isokinetic tests of absolute (W) and relative (W·kg^−1^) muscle power, maximal and endurance. The statistical method (SM) was parametric (P) or non-parametric (NP) (see Methods section). Parametric tests are presented as mean ± standard deviation (min-max). Non-parametric tests are presented as median ± Interquartile range (min-max). Groups marked with different symbols in rows (*, †) are significantly (p<0.01) different (* significantly different from †).

The absolute maximal knee extension (p = 0.006), absolute endurance knee extension (p<0.0001), absolute endurance trunk flexion (p<0.0001) and absolute endurance trunk extension (p = 0.001) were significantly different between subject groups. All groups of men had higher power compared to the CW group in these tests, but groups of men had equal power (Table 4).

**Table 4:** Subject group (N = 38) performances in isokinetic concentric tests of lower body and trunk muscle power and postural stability.

		Firefighters		Civilians	
Test	SM	Full time	Part time	Men	Women
		N = 10	N = 8	N = 8	N = 12
**MAXIMAL MUSCLE POWER**					
**Knee extension**	NP	152±24.8 †	149±30.6 †	152±50.4 †	103±27.0 *
**(W)**		(133–209)	(105–209)	(98–186)	(84–125)
**Knee extension**	P	2.0±0.28 †	1.8±0.25 *†	1.8±0.30 *†	1.5±0.26 *
**(W·kg^−1^)**		(1.7–2.6)	(1.5–2.2)	(1.2–2.2)	(1.2–2.0)
**Knee flexion**	NP	91±13.9 †	76±27.6 *†	82±12.1 *†	63±16.3 *
**(W)**		(81–107)	(53–109)	(76–97)	(48–109)
**Knee flexion**	P	1.1±0.10	1.0±0.18	1.0±0.08	1.0±0.24
**(W·kg^−1^)**		(1.0–1.3)	(0.6–1.2)	(0.9–1.2)	(0.6–1.4)
**ENDURANCE MUSCLE POWER**					
**Knee extension**	P	215±32.6 †	208±38.0 †	210±32.8 †	146±27.2 *
**(W)**		(160–280)	(163–280)	(174–276)	(108–197)
**Knee extension**	P	2.7±0.37 †	2.6±0.34 *†	2.5±0.25 *†	2.1±0.40 *
**(W·kg^−1^)**		(2.1–3.4)	(2.0–3.0)	(2.1–2.8)	(1.6–2.8)
**Knee flexion**	NP	120±39.7	106±31.3	105±29.0	88±21.2
**(W)**		(77–143)	(74–138)	(87–121)	(65–155)
**Knee flexion**	NP	1.6±0.30	1.5±0.43	1.3±0.72	1.3±0.29
**(W·kg^−1^)**		(1.3–1.7)	(0.9–1.7)	(1.1–1.8)	(1.0–2.3)
**Deadlift**	P	1140±178.1 †	1046±290.4 *†	1083±185.7 *†	762±232.1 *
**(W)**		(942–1503)	(521–1391)	(904–1401)	247–1148)
**Deadlift**	P	14.4±2.1	12.6±2.5	13.1±2.2	11.1±3.4
**(W·kg^−1^)**		(11.8–19.0)	(7.2–15.2)	(10.3–16.2)	(2.5–15.9)
**Trunk flexion**	P	110±14.4 †	112±21.6 †	102±12.9 †	68±10.4 *
**(W)**		(87–138)	(85–154)	(88–124)	(57–94)
**Trunk flexion**	P	1.4±0.18 †	1.4±0.21 †	1.2±0.11 *†	1.0±0.15 *
**(W·kg^−1^)**		(1.1–1.7)	(1.1–1.7)	(1.1–1.4)	(0.7–1.3)
**Trunk extension**	NP	260±61.3 †	245±54.0 †	264±139.5 †	165±29.1 *
**(W)**		(191–335)	(194–328)	(177–393)	(135–260)
**Trunk extension**	NP	3.5±0.96	3.0±0.68	3.0±1.5	2.5±1.7
**(W·kg^−1^)**		(2.2–4.2)	(2.7–3.5)	(2.6–4.6)	(1.5–3.3)
**DYNAMIC BALANCE**					
**Postural stability**	NP	3.5±0.8 †	3.3±3.0 *†	4.1±4.3 *†	1.9±0.7 *
**(SI)**		(2.1–3.9)	(2.2–9.4)	(1.4–10.2)	(0.9–4.1)

Isokinetic tests of absolute (W) and relative (W·kg^−1^) muscle power, maximal and endurance. The statistical method (SM) was parametric (P) or non-parametric (NP) (see Methods section). Parametric tests are presented as mean ± standard deviation (min-max). Non-parametric tests are presented as median ± Interquartile range (min-max). Groups marked with different symbols in rows (*, †) are significantly (p<0.01) different (* significantly different from †).

Relative maximal (p<0.0001) and endurance (p = 0.004) knee extension, absolute maximal knee flexion (p = 0.001), and absolute endurance deadlift power (p = 0.002) were significantly different between subject groups. The MFF group had higher power compared to the CW group in these tests but no significant differences were found between the other subject groups (Table 4).

Relative endurance trunk flexion power (p<0.0001) was higher for groups of firefighters compared to the CW group, and without significant differences between the male groups, and between the CM and the CW group (Table 4).

No significant differences were found between subject groups in relative maximal (p = 0.064) and endurance knee flexion (p = 0.115), relative endurance deadlift (p = 0.062), and relative endurance trunk extension power (p = 0.03). The Kruskal-Wallis test indicated significant differences between subject groups for absolute endurance knee flexion power (p = 0.006), but no significant differences were found in the post hoc analyses (Table 4).

The dynamic balance was significantly different between subject groups (p = 0.003): the CW group had higher dynamic balance (lower SI) compared to the MFF group, and no significant differences were found between the other subject groups (Table 4).

#### Field tests

All groups of men reached a higher maximal hand grip strength, endurance hand grip time, number of bench presses, number of chin-ups, number of dips, and standing broad jump length compared to the CW group (p<0.0001), but no significant differences were found between the male groups in these tests (Table 5). The MFF group completed a higher number of sit-ups compared to the CM and CW groups (p = 0.001), no significant differences were found between the other subject groups in the sit-up test (Table 5). The MFF group completed a higher number of squats and upright barbell rows (p<0.0001) compared to all the other subject groups, and no significant differences were found between the other subject groups in these two tests (Table 5). The number of barbell shoulder presses performed was higher for the MFF group compared to the CW group (p<0.0001), but no significant differences were found between the male groups, or between the MPF, CM and CW groups in the barbell shoulder press test (Table 5).

**Table 5:** Subject group (N = 38) performances in field tests and simulated work tasks.

		Firefighters		Civilians	
Test	SM	Full time	Part time	Men	Women
		N = 10	N = 8	N = 8	N = 12
**FIELD TESTS**					
**Maximal hand grip strength**	P	61±6.6 †	63±7.8 †	63±10.8 †	36±3.6 *
**(Kg)**		(52–75)	(56–79)	(43–71)	(32–43)
**Sit-ups**	NP	162±164 †	95±44 *†	56±12 *	48±31 *
**(N)**		(66–500)	(32–114)	(50–68)	(27–101)
**Endurance hand grip**	NP	271±159.3 †	201±99.5 †	221±106.8 †	87±30.8 *
**(s)**		(133–420)	(144–312)	(170–377)	(45–257)
**Squat**	P	118±40.5 †	65±23.9 *	60±17.8 *	43±22.0 *
**(N)**		(50–168)	(36–100)	(39–91)	(15–80)
**Bench press**	NP	57±14 †	53±28 †	40±31 †	2±3 *
**(N)**		(23–77)	(38–77)	(19–60)	(0–28)
**Chin-ups**	NP	15±14 †	11±6 †	9±10 †	0±0 *
**(N)**		(6–22)	(5–17)	(2–15)	(0–3)
**Dips**	NP	28±17 †	22±10 †	19±19 †	0±2 *
**(N)**		(0–35)	(12–40)	(3–30)	(0–16)
**Upright barbell row**	P	212±75.1 †	105±43.1 *	110±35.9 *	74±27.3 *
**(N)**		(103–310)	(54–175)	(59–162)	(43–125)
**Standing broad jump^ a^**	P	249±14.6 †	248±17.0 †	243±37.2 †	189±23.4 *
**(m)**		(230–269)	(226–272)	(164–295)	(143–226)
**Barbell shoulder press**	NP	124±40 †	87±27 *†	72±54 *†	50±21 *
**(N)**		(55–177)	(66–114)	(45–134)	(34–101)
**SIMULATED WORK TASK**					
**Cutting ^a^**	P	423±232	351±187	351±105	188±57
**(s)**		(173–900)	(142–598)	(186–512)	(115–265)
**Stairs ^b^**	P	65±8.2 †	79±14.6 †	78±19.4 †	188±89.9 *
**(s)**		(51–82)	(61–106)	(53–111)	(95–374)
**Pulling ^a^**	P	15±2.5 †	14±2.1 †	19±3.5 †	33±9.6 *
**(s)**		(11–19)	(11–17)	(15–24)	(19–49)
**Demolition ^a^**	P	53±11.8 †	47±7.7 †	45±8.8 †	20±13.4 *
**(s)**		(30–72)	(37–58)	(36–58)	(1–44)
**Rescue ^a^**	P	19±3.5 †	19±3.2 †	22±5.1 †	32±6.0 *
**(s)**		(16–25)	(14–24)	(17–31)	(23–40)
**Terrain**	NP	645±103 †	674±59 *†	683±63 †	885±217 *
**(s)**		(528–716)	(630–915)	(609–786)	(693–1074)

The statistical Method (SM) was parametric (P) or non-parametric (NP) (see Methods section). Parametric tests are presented as mean ± standard deviation (min-max). Non-parametric tests are presented as median ± Interquartile range (min-max). Groups marked with different symbols in rows (*, †) are significantly (p<0.01) different (* significant different from †). Investigated work tasks were: Carrying hose baskets in a staircase (Stairs), Hose pulling (Pulling), Demolition at or after a fire (Demolition), Victim rescue (Rescue), and Carrying hose baskets over terrain (Terrain). Data is presented in kilograms (kg), Number of repetitions (N), or seconds (S). ^a^ One CW subject was excluded ^b^ Two CW subjects were excluded.

#### Simulated work tasks capacity

Due to the large number of subjects reaching maximal time [n = 33 (87%)], the *Vehicle* task was removed from further data analysis. No significant differences were seen between subject groups performance in the *Cutting* task (p = 0.014) (Table 5). Significant group differences were found in the *Stairs, Pulling, Demolition* and *Rescue* work tasks (p<0.0001 for all these tasks): all groups of men reached higher performance compared to the CW group, and no significant differences were found between the male subject groups (Table 5). The *Terrain* (p = 0.001) task was executed faster by the MFF and CM groups compared to the CW group, and without significant differences between the other subject groups (Table 5). No differences were found between groups in the average mean (± SD) (min and max) % HR_max_ (84±4% (75% and 91%)) or the highest mean % HR_max_ (90±4.5% (81% and 98%) achieved during the work task course. Neither was any differences found between subject groups in the average mean % HR_max_ (89±5% (78% and 96%)) or the highest mean % HR_max_ (95±3.9% (88% and 100%) achieved during the *Terrain* work task.

### Correlations

#### Laboratory tests versus simulated work tasks

All laboratory tests of maximal and endurance (absolute and relative) upper body muscle power were significantly correlated (r_s_ = −0.83 to 0.53) with work capacity time in the *Stairs, Pulling, Demolition* and *Rescue* work tasks (Table 6). The *Cutting* work task time was significantly correlated (r_s_ = 0.41 to 0.58) with performance in all Isokinetic tests in the upper the body, except the absolute endurance shoulder press test (r_s_ = 0.38, p = 0.020), and the relative endurance shoulder extension test (r_s_ = 0.38, p = 0.019) (Table 6). *Terrain* work capacity was significantly correlated (r_s_ = −0.44 to −0.56) with all tests of absolute and relative muscle power in the upper the body, except the absolute and relative endurance shoulder press test (r_s_ = −0.37 and −0.33, p = 0.024 and 0.04, respectively) (Table 6).

**Table 6:** Correlations between work task capacity and laboratory tests of absolute and relative muscle power in the upper body.

	Cutting (s)^a^	Stairs(s)^b^	Pulling (s)^a^	Demo (s)^a^	Rescue (s)^a^	Terrain (s)
**E. Shoulder press (W)**	0.38	−0.73^*^	−0.73^*^	0.63^*^	−0.77^*^	−0.37
**E. Shoulder press (W·kg^−1^)**	0.41^*^	−0.70^*^	−0.58^*^	0.53^*^	−0.64^*^	−0.33
**M. Shoulder flexion (W)**	0.53^*^	−0.79^*^	−0.72^*^	0.68^*^	−0.81^*^	−0.47^*^
**M. Shoulder flexion (W·kg^−1^)**	0.58^*^	−0.75^*^	−0.60^*^	0.61^*^	−0.69^*^	−0.56^*^
**M. Shoulder extension (W)**	0.48^*^	−0.71^*^	−0.79^*^	0.64^*^	−0.78^*^	−0.49^*^
**M. Shoulder extension (W·kg^−1^)**	0.41^*^	−0.56^*^	−0.59^*^	0.47^*^	−0.57^*^	−0.44^*^
**E. Shoulder flexion (W)**	0.48^*^	−0.80^*^	−0.78^*^	0.69^*^	−0.83^*^	−0.48^*^
**E. Shoulder flexion (W·kg^−1^)**	0.49^*^	−0.81^*^	−0.69^*^	0.68^*^	−0.74^*^	−0.56^*^
**E. Shoulder extension (W)**	0.46^*^	−0.71^*^	−0.82^*^	0.65^*^	−0.80^*^	−0.48^*^
**E. Shoulder extension (W·kg^−1^)**	0.38	−0.56^*^	−0.68^*^	0.52^*^	−0.62^*^	−0.46^*^

Spearman's rank correlation coefficient (r_s_) was used to analyze the simulated work tasks correlation with maximal (M) and endurance (E) Isokinetic tests. Isokinetic tests are analyzed with the mean absolute (W) and relative (W·kg^−1^) power. Investigated work tasks were: Cutting holes in the roof for fire gas ventilation (Cutting), Carrying hose baskets in a staircase (Stairs), Hose pulling (Pulling), Demolition at or after a fire (Demo), Victim rescue (Rescue), and Carrying hose baskets over terrain (Terrain), with time in seconds (s) used in the analyzes. ^*^p<0.01. Subjects N = 38 ^a^ One CW subject was excluded ^b^ Two CW subjects were excluded. Numbers in bold types indicate the laboratory test with the highest correlation with each work task.

All simulated work tasks were significantly correlated (r_s_ = −0.78 to −0.41) with at least nine (out of 14) performance results from isokinetic absolute and relative muscle power in the lower body and trunk, the significant correlations ranged between (Table 7). The relative maximal and endurance knee flexion and the relative endurance deadlift power were without significant correlation (p = 0.019 to 0.256) with all work tasks (Table 7).

**Table 7:** Correlations between work task capacity and laboratory tests: absolute and relative muscle power in the lower body and the trunk, and dynamic balance.

	Cutting(s)^a^	Stairs (s)^b^	Pulling (s)^a^	Demo (s)^a^	Rescue (s)^a^	Terrain (s)
**M. Knee extension (W)**	0.61^*^	−0.78^*^	−0.65^*^	0.59^*^	−0.78^*^	−0.41^*^
**M. Knee extension (W·kg^−1^)**	0.56^*^	−0.60^*^	−0.36	0.37	−0.53*	−0.32
**M. Knee flexion (W)**	0.59^*^	−0.66^*^	−0.55^*^	0.59^*^	−0.61^*^	0.51^*^
**M. Knee flexion (W·kg^−1^)**	0.38	−0.33	−0.19	0.28	−0.27	−0.36
**E. Knee extension (W)**	0.51^*^	−0.76^*^	−0.69^*^	0.75^*^	−0.78^*^	−0.54^*^
**E. Knee extension (W·kg^−1^)**	0.43^*^	−0.61^*^	−0.42^*^	0.55^*^	−0.54^*^	−0.48^*^
**E. Knee flexion (W)**	0.44^*^	−0.53^*^	−0.53^*^	0.58^*^	−0.58^*^	−0.53^*^
**E. Knee flexion (W·kg^−1^)**	0.24	−0.24	−0.23	0.29	−0.30	−0.38
**E. Deadlift (W)**	0.48^*^	−0.46^*^	−0.62^*^	0.51^*^	−0.56^*^	−0.43^*^
**E. Deadlift (W·kg^−1^)**	0.34	−0.15	−0.34	0.20	−0.19	−0.33
**E. Trunk extension (W)**	0.72^*^	−0.68^*^	−0.58^*^	0.55^*^	−0.77^*^	−0.47^*^
**E. Trunk extension (W·kg^−1^)**	0.71^*^	−0.50^*^	−0.28	0.30	−0.52^*^	−0.35
**E. Trunk flexion (W)**	0.47^*^	−0.74^*^	−0.77^*^	0.69^*^	−0.76^*^	−0.55^*^
**E. Trunk flexion (W·kg^−1^)**	0.44^*^	−0.61^*^	−0.59^*^	0.56^*^	−0.55^*^	−0.58^*^
**Dynamic balance (SI)**	0.42^*^	−0.45^*^	−0.53^*^	0.42^*^	−0.51^*^	−0.36

Spearman's rank correlation coefficient (r_s_) was used to analyze the simulated work tasks correlation with maximal (M) and endurance (E) Isokinetic tests, and dynamic balance. Isokinetic tests are analyzed with the mean absolute (W) and relative (W·kg^−1^) power and dynamic balance is analyzed with is analyzed with the overall stability index (SI). Investigated work tasks were: Cutting holes in the roof for fire gas ventilation (Cutting), Carrying hose baskets in a staircase (Stairs), Hose pulling (Pulling), Demolition at or after a fire (Demo), Victim rescue (Rescue), and Carrying hose baskets over terrain (Terrain), with time in seconds (s) used in the analyzes. ^*^p<0.01. Subjects N = 38 ^a^ One CW subject was excluded ^b^Two CW subjects were excluded. Numbers in bold types indicate the laboratory test with the highest correlation with each work task.

The highest correlations with the *Cutting, Demolition*, and *Terrain* work tasks were found with lower body and trunk muscle power. *Cutting* had the highest correlation with absolute endurance trunk extension (r_s_ = 0.72), *Demolition* had the highest correlation with absolute endurance knee extension (r_s_ = 0.75), and the *Terrain* work task had the highest correlation with relative endurance trunk flexion power (r_s_ = −0.58) (Table 7). The highest correlations with the *Stairs, Pulling*, and *Rescue* work tasks were found with upper body muscle power. *Stairs* had the highest correlation with relative endurance shoulder flexion (r_s_ = −0.81), *Pulling* had the highest correlation with absolute endurance shoulder extension (r_s_ = −0.82), and the *Rescue* work task had the highest correlation with absolute endurance shoulder flexion power (r_s_ = −0.83) (Table 6).

Dynamic balance was significantly correlated with all simulated work tasks (r_s_ = 0.42 to −0.53) except the *Terrain* work task (r_s_ = −0.36, p = 0.026).

#### Field tests versus simulated work tasks

All field tests were significantly correlated (r_s_ = −0.85 to 0.40) with work capacity time in all work tasks. In general, the highest correlations with work time on the tasks were found for upper body muscle strength and endurance: *Pulling, Demolition*, and *Rescue* had the highest correlations with the number of bench presses performed (r_s_ = −0.85, 0.83, and −0.82, respectively). *Cutting* had the highest correlation (r_s_ = 0.67) with maximal hand grip strength, *Stairs* had the highest correlation (r_s_ = −0.77) with the number of barbell shoulder presses performed, and *Terrain* had the highest correlation (r_s_ = −0.70) with upright barbell row (Table 8).

**Table 8:** Correlations between work task capacity and field tests.

	Cutting(s)^a^	Stairs (s)^b^	Pulling (s)^a^	Demo(s)^a^	Rescue (s)^a^	Terrain (s)
**Maximal hand grip strength (kg)**	0.67	−0.69	−0.73	0.66	−0.79	−0.5
**Sit ups (N)**	0.50	−0.56	−0.51	0.51	−0.44	−0.47
**Endurance hand grip (s)**	0.59	−0.59	−0.67	0.62	−0.68	−0.62
**Squat (N)**	0.42	−0.63	−0.57	0.56	−0.59	−0.56
**Bench press (N)**	0.47	−0.73	−**0.85**	**0.83**	−**0.82**	−0.56
**Chin ups (N)**	0.54	−0.76	−0.72	0.69	−0.74	−0.53
**Dips (N)**	0.50	−0.75	−0.61	0.69	−0.81	−0.53
**Upright barbell row**	0.55	−0.66	−0.62	0.74	−0.65	−**0.70**
**Standing broad jump (m)** ***^a^***	0.40	−0.72	−0.67	0.53	−0.74	−0.47
**Barbell shoulder press (N)**	0.55	−0.77	−0.66	0.70	−0.76	−0.65

Spearman's rank correlation coefficient (r_s_) was used to analyze the simulated work tasks correlation with field tests. Investigated work tasks were: Cutting holes in the roof for fire gas ventilation (Cutting), Carrying hose baskets in a staircase (Stairs), Hose pulling (Pulling), Demolition at or after a fire (Demo), Victim rescue (Rescue), and Carrying hose baskets over terrain (Terrain). Data used in the analyses are seconds (s), kilograms (kg), Number (N), or meters (m). All field tests were significantly correlated with all simulated work tasks (p<0.01). Subjects N = 38 ^a^ One CW subject was excluded ^b^Two CW subjects were excluded. Numbers in bold types indicate the field test with the highest correlation to each simulated work task. ^a^ One CW subject was excluded ^b^Two CW subjects were excluded. Numbers in bold types indicate the laboratory test with the highest correlation with each work task.

## Discussion

To our knowledge, this is the first study investigating correlations of firefighters work capacity, using both laboratory and field tests. The main finding is that several laboratory tests and all field tests included in the present study are significantly correlated with firefighters work capacity, and field tests can be used instead of laboratory tests. Maximal hand grip strength, bench press, chin-ups, dips, standing broad jump, upright barbell row and barbell shoulder press all have strong correlations (r_s_≥0.7) with at least one simulated work tasks, and might be the most relevant physical tests for evaluation of firefighters' work capacity.

### Physical performance

#### Laboratory and field tests

As a group, and as expected, women had lower levels of physical performance than all three groups of men in several laboratory tests and field tests, both in the upper and the lower body [47], [48], but they had higher dynamic balance compared to the MFF group. Ten and seven CW subjects were not able to perform one single repetition of chin-ups or dips, respectively. It is unknown if subjects in the CW group were engaged in upper-body strength training to the same extent as the male groups. If not, specific training would probably result in higher performance more in line with that of the male groups [49]. Men as a group, have more skeletal muscle mass compared to women, both in absolute terms and in relation to body mass, with the greatest difference being in the upper body [50]. Also, Flanagan et al. [51] concluded that trained women have a higher bench press performance (85% 1 RM) than men when a muscle contraction is isolated concentric or eccentric, but the opposite conditions prevails between sexes in combined concentric and eccentric muscle contractions. The author concluded that these differences between sexes might be caused by a greater reliance on the stretch-shortening cycle among men [51].

Previous studies have found that postural stability and balance are affected by several factors among healthy subjects, including gender [52], BMI [53], and age (Biodex Balance system SD: Operation manual), with men having lower stability (higher SI values) compared to women. The SI index for all subject groups included in the present study ranged within normal values in relation to age and gender (Biodex Balance system SD: Operation manual), and no significant differences were found between subject groups in age and BMI.

#### Work capacity

Firefighting work is physically strenuous and the muscular demands of the work have previously been classified as medium to very heavy [54]. As a group, and as expected [6], [26], [28], [55], the work capacity of the CW group was lower on several of the simulated work tasks compared to men, and physiological differences between the sexes might be one important variable affecting work capacity. However, some women performed better than some men on all tests indicating that none of the included simulated work tasks were discriminative based on sex. Because of the inclusion of civilian's in the present study, an important question is whether the technique affected work capacity. The CM group had equal work capacity as groups of firefighters (Table 5), showing that the lack of experience did not affect the outcome and demonstrating that the present study investigated work capacity rather than work performance.

### Correlations

It has been suggested that simulated work tasks have a high validity for measuring firefighters' work capacity and that simulated work tasks do not measure fitness [49]. Inclusion of laboratory tests among firefighters has previously been requested by Barr et al.[3]. In line with Sothmann et al. [6] and Henderson et al. [33], we agree that simple field tests should be advocated if the purpose is to investigate physical fitness because laboratory tests of muscle performance are complicated, time consuming, and expensive. If the purpose is to investigate work performance, simulated work tasks might be more relevant.

Spearman r_s_ indicates the strength between two variables, but correlations does not necessary imply causation and we cannot conclude that one variable causes the other, although it is possible [56].

#### Laboratory tests versus work tasks

The repeatability of Isokinetic testing is high [57] when the tests are performed accurately and windowing is used [41]. One of the problems with Isokinetic testing is that the movement is isolated and not functional. Only performing concentric muscle contractions does not imitate a real time work situation. Because of that, the face validity (the obvious measurement with the tests [58]) is questionable for isolated isokinetic testing. A real time firefighting work includes both concentric and eccentric muscle work often performed over several joints simultaneously. We have not found any study investigating correlations between Isokinetic tests and firefighters work capacity. Barnekow-Bergkvist et al. [1] found maximal Isokinetic knee flexion and shoulder extension and Isometric back endurance to be significantly correlated with carrying a 94 kg stretcher, among ambulance personnel. In the present study, strong correlations with performance in at least one Isokinetic test were found for all simulated work tasks except the *Terrain* work task. Other capacities, such as aerobic power [27] might be more important for the *Terrain* work task. Although we found strong correlations between Isokinetic tests and work task capacities, these results only gives a glimpse of the complexity of valid physical laboratory testing of firefighters. Isokinetic testing is more commonly used within sports, and varying results are presented. For example rowing performance [59], aerobic power, and peak work rate on a cycle ergometer [60] is significantly correlated with Isokinetic knee extension performance. However, Carlsson et al. [61] did not find cross-country skiing performance among male elite cross-country skiers to be significantly correlated with Isokinetic knee extension performance.

#### Field tests versus work tasks

All work tasks but the *Cutting* work capacity time were strongly correlated with performance in a minimum of one and a maximum of six field tests. According to the Dusick reference values [46] moderate and moderate to low correlations were found with the *Cutting* work capacity. Within all these work tasks except the *Cutting* task, aerobic power is also of vital importance [27].

Both upper- and lower body muscular performance is important for firefighters' work capacity as previously suggested by others [6], [26], [30]–[35]. The strong and moderate correlations found between firefighters work capacity and maximal hand grip strength in the present study (Table 8), are in line with others [26], [30], [33]–[35]. In accordance with Philips et al. [35] but contrary to Rhea et al. [30], we found endurance hand grip to be moderately significantly correlated with work capacity (Table 8). The methods were different: the present study used a 27 kg barbell and the others [30], [35] used a dynamometer with the subjects aiming to maintain a 25 kg pressure as long as possible.

In accordance with others [30], [31], [34], the present study found endurance bench press to be significantly correlated with firefighters' work capacity although the methods used are different. Bench press performance was strongly correlated with all investigated work tasks except *Cutting* and *Terrain* (Table 8). Interestingly, Sheaff et al. [26] did not find significant correlation between firefighters' work capacity and endurance bench press performance. However, the methods within this and Sheaff's study were different. Sheaff et al. [26] used an air-powered resistance training-machine, with subjects having different loads, based on the 1RM bench press.

We have not found any study investigating chin-ups and dips performance in firefighters. In the present study, these tests were strongly correlated with *Stairs*, *Pulling*, and *Demolition* work capacity (Table 8). Due to the risk of discrimination against women, the low median performance within the CW group in these indirect tests makes it questionable whether they should be included among the end-point tests for firefighters' work capacity. Instead, other tests than chin-up and dips should be used to investigate upper body muscle strength and endurance.

In accordance with Rhea et al. [30], and in contrast to Philips et al. [35], we found shoulder press to be significantly correlated with work capacity and to be strongly correlated with the *Stairs, Demolition*, and *Rescue* tasks (Table 8), but both Rhea et al. [30] and Philips et al. [35] used a heavier barbell weight.

Lower body muscle power [32], [35], maximal muscle strength [34], and muscle endurance [30] has previously been found to be significantly correlated with firefighters work capacity. Rhea et al. [30] used a heavier barbell (61.4 kg) during the endurance squat test, compared to the present study (22.0 kg). Although the methods were different, both the present study and Rhea et al. [30] found significant correlations with firefighters work capacity. These results indicate that testing firefighters leg muscle strength, endurance – or power is of vital importance. Standing broad jump is testing lower body muscle power [35], and power is a product of a muscles force and speed [62].

In accordance with Michaelides et al. [34] but contrary to Rhea et al. [30], we found significant correlations between work capacity and sit-ups. According to the Dusick reference values [46], Michaelides et al. [34] found weak and low correlations and the present study found moderate and low correlations with the sit-ups test. However, Michaelides et al. [34] had a larger subject group inclusion (N = 90) than the present study, and comparisons between the studies are not accurate.

#### The plethora of tests

The kinds of field tests previously used to investigate firefighters' work capacity have been subject to considerable variation, and only a few of these tests were included in the present study. No other study but the present has investigated Isokinetic tests correlation with firefighters work capacity. Field tests of maximal handgrip strength [26], [30], [33]–[35], sit-ups [30], [31], [33], [34], handgrip endurance [30], [35], squat [30], bench press [26], [30], [31], [33], [34], standing broad jump [32], [35], and shoulder press [30], [35] have previously been used although the methods for performing the tests were often different from the present study. In accordance to Rhea et al. [30], the present study used predefined loads for endurance squat, shoulder press, and bench press, but Rhea et al. [30] used heavier barbell weights.

Examples of previously used field tests that were not included in the present study are 1 RM squat [34], leg press and leg extension [26], 1 RM bench press [31], [34], and calculation of 1 RM [33], 5 RM, and 80% of 1 RM bench press [26].

Due to the large variations in test methods between studies, comparing results is difficult. In a real-life situation, firefighters are expected to do the same work and this makes it reasonable to use predefined loads in the experimental tests. The disadvantage with using predefined loads is that the same test might be an endurance test for some subjects and a test of maximal muscle strength for others (e.g. bench press in the present study (Table 5)). Work tasks included in the present study are quite similar to those in previous studies [6], [26], [30], [31], [34] except for the pack hike test [35]. The pack hike test measures performance over a 4.83 km hike wearing a 20.4 kg load, and the work time is longer than all work tasks included in the present study [35]. This work task was not included because it was not regarded as commonly occurring among Swedish firefighters. The included *Vehicle* and *Cutting* tasks have not previously been investigated as described in the present study.

Firefighters' work performance is not affected only by physical fitness [11]–[14], [17], and tests of work performance are often biased due to factors other than physical fitness. If the purpose is to analyze correlations between firefighters' physical fitness and work capacity by using both simulated work tasks and fitness tests, as in the present and in previous studies [6], [26], [30]–[32], [34], [35], it is desirable to exclude or reconfigure work tasks that require a significant amount of experience or in other ways might affect the results. For example, Henderson et al. [33] included ladder climbing in the work tasks course, and with this task other factors, such as acrophobia, might affect the results.

### Limitations

There are only a few female firefighters working within the Swedish fire and rescue service, and none could be recruited for this study. The lack of participating females resulted in unknown performance variables for female firefighters and prevented accurate comparison to males. Several studies investigating correlations between results on physical performance tests and firefighting work tasks include male subjects only [31], [34], [35] or, as in the present study, merge results from men and women [6], [26], [30], [33]. Consequently, no published study has determined if there are different limiting factors for men and women in firefighting work capacity. Using larger subject groups, and including more women in future studies can investigate this.

Punakallio [36], [37] concluded that poor balance reduces the perceived work ability, and that a poor dynamic stability increases the risk of slipping and falling. Unfortunately, no specific field test of postural stability or balance was included in the present study. Significant correlations between simulated work capacity and laboratory dynamic balance were found, and further investigations are necessary to investigate this relationship.

The design of the *Vehicle*, and *Cutting* work tasks were not satisfying. Still, it is important also to report not satisfying methods in order to increase the research knowledge within this area.

## Conclusion

Firefighters work capacity is significantly correlated with both laboratory tests and field tests. Using simple field tests should be advocated due to their simplicity, lower cost and time savings. Due to the strong correlations with firefighters work capacity, recommended field tests for evaluation of firefighters' work capacity are maximal hand grip strength, bench press, chin ups, dips, upright barbell row, standing broad jump, and barbell shoulder press.
